# Boosting angiogenesis experimentally in ovo by biofunctionalizing collagen membranes with platelet-rich fibrin and hyaluronic acid: implications for regenerative oral surgery?

**DOI:** 10.1186/s40729-026-00669-3

**Published:** 2026-01-17

**Authors:** Saskia-Vanessa Schröger, Johanna Becker, Sebahat Kaya, Roman Rahimi-Nedjat, Keyvan Sagheb, Sebastian Blatt

**Affiliations:** Department of Oral and Maxillofacial Surgery — Plastic Operations, University Medical Center Mainz, Augustusplatz 2, 55131 Mainz, Germany

**Keywords:** Platelet-rich plasma, Fibrin hyaluronic acid, Collagen, Chorioallantoic membrane, Angiogenesis, Neovascularization

## Abstract

**Purpose:**

Biocompatible collagen membranes (CM) are widely used in regenerative dentistry, particularly in guided tissue regeneration (GTR) and guided bone regeneration (GBR). While resorbable CMs offer advantages such as reduced patient morbidity and enhanced wound healing, their barrier function can impede vascularization, potentially compromising graft survival. Biofunctionalization of CMs with platelet-rich fibrin (PRF) and hyaluronic acid (HA) has shown promise in enhancing angiogenesis. This experimental study evaluates the pro-angiogenic effects of biofunctionalizing collagen membranes with advanced PRF (A-PRF), injectable PRF (i-PRF), and HA using the chorioallantoic membrane (CAM) assay in ovo.

**Methods:**

Three porcine-derived collagen membranes (Mucoderm®, Bio-Gide®, and Smartbrane®) were biofunctionalized with A-PRF, i-PRF, or HA and applied to the CAM assay on day 7 of incubation. Afterwards, two experimental series were evaluated. The first series investigated Mucoderm (MM native, A-PRF, and MM combined with A-PRF) over an observation period ranging from 24 to 120 h (*N* = 135). The second series examined Bio-Gide® and Smartbrane (BM native, BM combined with i-PRF, BM combined with HA, and SM combined with HA) over a period from 24 to 72 h (*N* = 60). To assess impact on angiogenesis, vascularization was evaluated at multiple time points (24 h, 48 h, 72 h, 96 h, and 120 h) using immunohistochemical staining (hematoxylin-eosin, α-smooth muscle actin, CD105) and artificial intelligence (AI)-assisted image analysis (IKOSA® software).

**Results:**

Biofunctionalization of Mucoderm® with A-PRF significantly enhanced angiogenesis up to 96 h, as evidenced by increased vessel area, length, and branching points (*p* < 0.05). I-PRF biofunctionalization of Bio-Gide® also promoted angiogenesis between 24 h (*p* = 0.036) and 72 h, showing significantly improved values for total area (*p* = 0.007), vessel length (*p* = 0.018), and vessel thickness (*p* = 0.008) compared with the native membrane. While HA biofunctionalization of Bio-Gide® and Smartbrane® resulted in significantly increased angiogenesis at 48 and 72 h (*p* < 0.05), its effects were less pronounced than those achieved with PRF variants. Native Bio-Gide® exhibited greater pro-angiogenic potential than native Mucoderm® at 24 h (*p* = 0.012); however, biofunctionalized membranes generally outperformed native variants.

**Conclusions:**

Biofunctionalization of collagen membranes with A-PRF and i-PRF significantly enhances angiogenesis in ovo, with A-PRF showing sustained effects up to 96 h. HA also promotes angiogenesis and represents a viable, cost-effective alternative that does not require blood collection. Both PRF and HA biofunctionalization may offer potential benefits for enhancing vascularization in GBR/GTR applications. However, their pro-angiogenic potential and clinical relevance remain exploratory at this stage for pourely in-ovo results. The study is limited to an in-ovo setting, therefore the transferability to oral soft tissue and bone conditions is restricted. Further randomized clinical studies are needed to better understand and evaluate the possible advantages.

## Introduction

Biocompatible collagen membranes (CM) are widely used in regenerative oral surgery, particularly in techniques such as guided tissue regeneration (GTR) and guided bone regeneration (GBR) [1]. These membranes support healing by helping to preserve the necessary stable space for tissue regeneration, allowing migration of periodontal and osteogenic precursor cells while blocking undesirable cells, such as fibroblasts [2]. More importantly, their function as space preservers and stabilizers prevents the collapse of soft tissue into the defect and facilitates the accumulation of growth factors [3]. Resorbable collagen membranes offer several clinical advantages. By eliminating the need for a second surgical procedure to remove the membrane, they reduce patient morbidity. If they are exposed, they can rapidly be resorbed, minimizing the risk of bacterial contamination and infection [4]. These membranes also support wound healing by promoting platelet adhesion, fibrin network formation, and the attachment of epithelial and connective tissue cells on the membranes surface [5]. In addition, they can absorb growth factors released by cells and bone, such as transforming growth factor-beta (TGF-β), thereby promoting bone regeneration.

However, clinical success depends on synchronizing the membrane’s resorption with the pace of tissues regeneration [6]—a significant challenge given the variable and often unpredictable resorption times of different materials [7]. Both premature and prolonged resorption can compromise outcomes in GBR [6, 8]. In addition, insufficient membrane stability may lead to collapse into the bone defect. To support bone regeneration, the concurrent use of autologous, allogenic, or xenogeneic bone grafts is recommended in clinical practice. While autologous grafts are particularly advantageous for bone regeneration due to their osteoconductive, osteoinductive, and osteogenic properties, they are associated with higher morbidity and rapid degradation within tissue [4]. A major limitation of resorbable (collagen) membranes is their barrier function, which can inhibit ingrowth of blood vessels. This decrease of vascularization can lead to insufficient or delayed blood supply, potentially compromising graft survival [9–11]. Efficient vascularization of the biomaterials is therefore crucial to ensure long-term survival and function [12]. Numerous studies have reported promising angiogenic effects upon biofunctionalization of CM via platelet-rich fibrin (PRF) [13, 14], an autologous platelet concentrate [15] that supports angiogenesis and tissue regeneration throughout all phases of the wound healing process and injectable-PRF (i-PRF) [16].

Hyaluronic acid (HA) is a naturally occurring glycosaminoglycan, composed of repeating disaccharide units of ß-D-glucuronic acid and N-acetyl-D-glucosamine [17, 18]. Its high biocompatibility, biodegradability, and hydrophilicity, along with non-immunogenicity and modifiability, make it attractive for medical use [19]. HA promotes wound healing by enhancing fibroblast adhesion, migration, and proliferation by stimulating collagen production [20]. Its functions also include reducing inflammation, regulating collagen remodeling and improving angiogenesis [21]—critical during the proliferative phase of healing [22, 23]. Koray et al. (2014) showed that HA spray reduced postoperative swelling and trismus after third molar extraction compared to benzydamine hydrochloride [24]. Current evidence suggests that hyaluronic acid may provide adjunctive benefits in periodontal and peri-implant therapy, including reduced inflammation. However, due to study heterogeneity and risk of bias, well-designed randomized trials are still needed [25]. A study by Kyyak et al. (2022) has demonstrated that combining a xenogeneic bone substitute material with HA significantly increases angiogenesis in vivo [26]. Additionally, the biofunctionalization of bovine bone substitute materials with HA has been reported to increase the activity of human osteoblasts, potentially promoting oral bone regeneration [27]. Combining collagen membranes with HA thus constitutes another promising approach for optimizing pro-angiogenic properties. This experimental study investigates the biofunctionalization of CM with A-PRF, i-PRF and HA and uses an *in ovo* model to assess its impact on vascularization and angiogenesis.

The CAM model offers several strengths relevant to oral bone regeneration research. It provides a highly vascularized and immunodeficient environment that allows rapid assessment of angiogenic responses to biomaterials such as collagen membranes, PRF, and hyaluronic acid. The model is quick, cost-effective, ethically less restrictive than animal experimental models, and enables high-throughput screening under controlled conditions [28, 29]. CAM assays generally involve placing a test material onto the highly vascular membrane, which readily accommodates and integrates diverse biomaterials [30]. Early work by Coleman and Garrison (1972) showed that CAM cells possess strong calcium-trafficking capacity [31–33]. Together with its rich blood supply and oxygenation, this provides three essential conditions for bone formation and helped establish the CAM as a useful experimental model for studying mandibular bone regeneration.

Despite these strengths, protocol variability can lead to inconsistent outcomes, and quantifying angiogenesis remains difficult due to high variability in visual and image-based assessments. The CAM also lacks a mineralized matrix, a mature immune system, and mechanical loading, limiting its comparability to human oral bone. Thus, it predominantly reflects early vascular responses rather than long-term bone formation or clinical performance [28, 30].

Overall, the CAM assay is a valuable exploratory model for early angiogenesis and biocompatibility testing, but results require confirmation in mammalian in vivo models before clinical translation. The aim of this study was to explore the angiogenic effects of collagen membranes biofunctionalized with PRF and HA in the CAM model. The novelty of this study lies on one hand in its extended observation period (up to 120 h) in ovo and on the other hand in the comparison of hyaluronic acid and platelet-rich fibrin for membrane biofunctionalization. We hypothesized that PRF- and HA-biofunctionalized membranes might enhance angiogenic responses compared with native membranes, PRF and HA could exhibit different angiogenic profiles, and lastly angiogenic activity may vary over time during the extended evaluation period.

By comparing the pro-angiogenic potential of different CM biofunctionalization approaches, this work aims to inform clinical decision-making in regenerative oral surgery.

## Materials and methods

See Fig. 1.

**Fig. 1:**
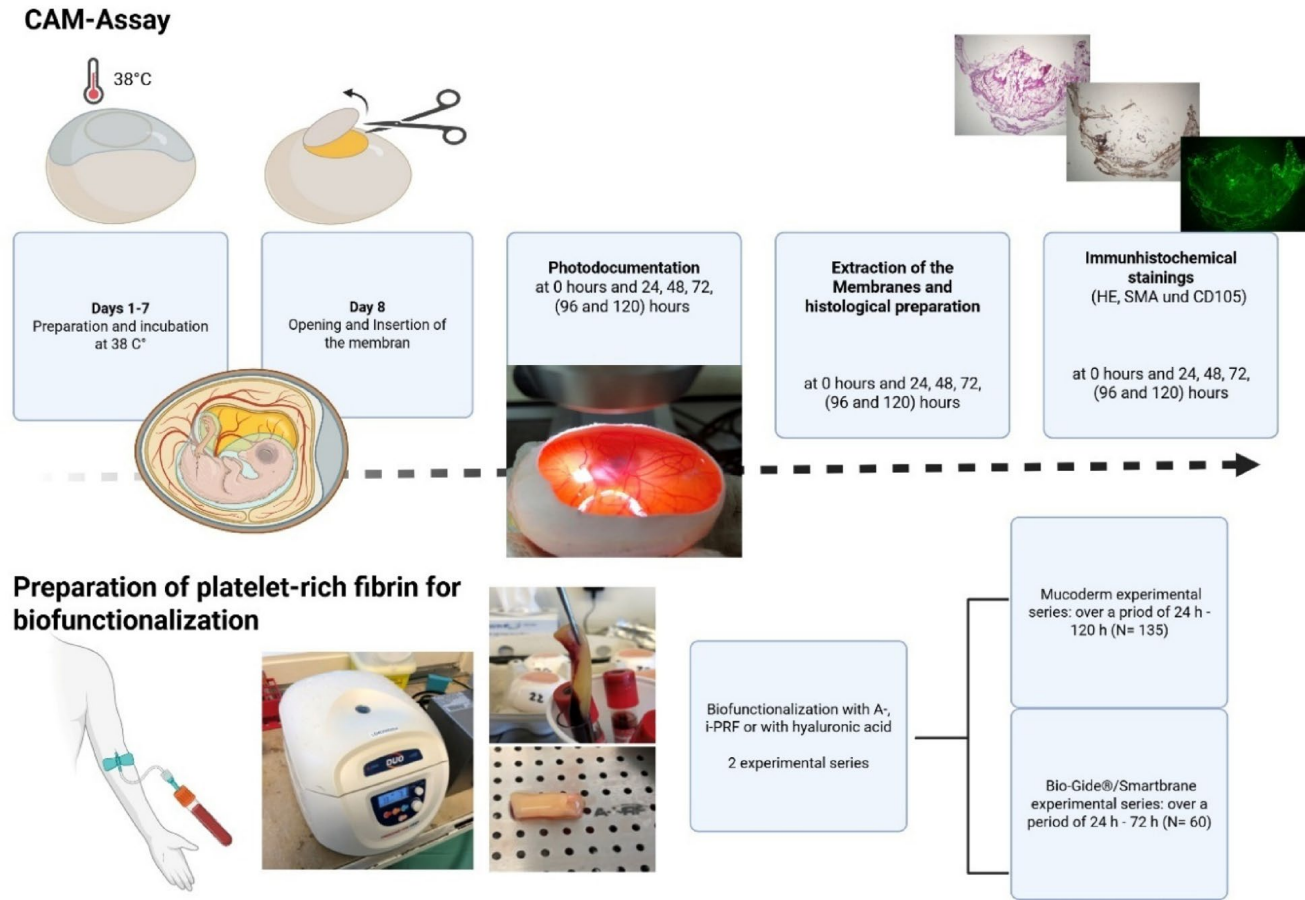
Workflow experimental design

### Membranes

In this study, the Mucoderm® membrane (Botiss, Zossen, Germany), Bio-Gide® membrane (Geistlich, Baden-Baden, Germany), and Smartbrane membrane (Regedent AG, Zurich, Switzerland) were analyzed. All membranes are composed of collagen and are derived from porcine sources.

The Mucoderm® membrane (Botiss, Zossen, Germany) is a collagen membrane approximately 1.2–1.7 mm thick, composed of type I and type III collagen. Derived from porcine dermis, the collagen matrix is free from artificial cross-linking and closely resembles the collagen structure of human skin. Its rough and porous surface facilitates the ingrowth of blood vessels and tissue cells, thereby promoting rapid vascularization and integration. To enhance its mechanical properties, the manufacturer recommends rehydration in saline solution or blood for 5–20 min [34]. The Mucoderm® membrane (Botiss, Zossen, Germany) was rehydrated for 10 min in a 0.9% saline solution (Merck, Darmstadt, Germany) in accordance with the manufacturer’s recommendations.

Bio-Gide® (Geistlich, Baden-Baden, Germany) is a bilayer collagen membrane derived from porcine type I and III collagen, free of artificial cross-linking. The smooth side of the membrane prevents the ingrowth of soft tissue cells into the defect, while the rough side provides a scaffold for the ingrowth of blood vessels and bone cells. Bio-Gide® is commonly used in clinical applications, including ridge preservation after tooth extraction, small and large bone augmentations, and sinus floor elevations. Its use has been shown to enhance bone regeneration and promote effective tissue integration [35, 36].

The Smartbrane membrane (Regedent AG, Zurich, Switzerland) is a native collagen membrane derived from porcine pericardium. The gentle cleaning process preserves the natural collagen matrix along with the intrinsic cross-linking of collagen fibers. With a thickness of less than 0.4 mm, the membrane is distinguished by its high tensile strength. It is suitable for use in guided bone regeneration (GBR) and guided tissue regeneration (GTR), including applications such as alveolar ridge augmentation, sinus lifts, alveolar chamber preservation following tooth extraction, and the treatment of periodontal bone defects [37]. All membranes were cut into squares of 20 mm^2^ (± 0.1 mm) under sterile conditions using a sterile scalpel.

### Preparation of platelet-rich fibrin and biofunctionalization

For the preparation of A-PRF, approximately 60 ml of venous blood was drawn from a healthy volunteer, following informed consent, using a specialized Vacutainer system (A-PRF+, Process for PRF, Nice, France). The blood was then centrifuged according to protocol at 1300 revolutions per minute (rpm) for 8 min (Duo centrifuge, Process for PRF, Nice, France). Subsequently, the PRF clot was separated from the remaining centrifuged blood using sterile scissors. The A-PRF was manually pressed for 60 s using a sterile PRF set (Process for PRF, Nice, France) and then cut into pieces measuring 20 mm^2^ (± 0.1 mm) under sterile conditions using a sterile scalpel. The blood collection process for i-PRF was conducted similarly, but i-PRF tubes (i-PRF, Process for PRF, Nice, France) were used. Centrifugation was performed at 700 rpm for 3 min as per the manufacturer’s instructions (Duo centrifuge, Process for PRF, Nice, France). The i-PRF was then aspirated under sterile conditions using a sterile syringe with a 21G needle (Becton Dickinson, Franklin Lakes, USA).

The Mucoderm® membrane was combined with the A-PRF by pressing them together using the PRF set (Process for PRF, Nice, France) for 30 s. For the biofunctionalization of the Bio-Gide® and Smartbrane membranes, a consistent amount of 1 ml hyaluronic acid (HyaDENT, Regedent AG, Zurich, Switzerland) was applied to the membranes just before placement. 1.0 ml of the HyaDENT hyaluronic acid contains 2.0 mg Hyaluronic acid (HyA), 16.0 mg of Hyaluronic acid cross-linked (xHyA), 6.9 mg Sodium chloride and 1.0 ml water. It is of non-animal origin and biocompatible. Additionally, the Bio-Gide® was biofunctionalized with i-PRF. The investigations in this study were conducted in accordance with the guidelines of the Declaration of Helsinki. Ethical approval was obtained from the Ethics Committee of the Medical Association of Rhineland-Palatinate (No. 2019–14705_1).

### Chorio-allantoic membrane assay

For these experimental series, fertilized Leghorn chicken eggs (LSL Rhein-Main, Dieburg, Germany) were used. Nine samples were included per group (no dropouts observed): native Mucoderm® membrane (MM native), Mucoderm® biofunctionalized with A-PRF (MM + A-PRF) and native A-PRF (A-PRF native) (in total *n* = 135), native Bio-Gide® (BM native), Bio-Gide® biofunctionalized with i-PRF (BM + i-PRF), Bio-Gide® biofunctionalized with hyaluronic acid (BM + HA), and Smartbrane membrane biofunctionalized with hyaluronic acid (SM + HA) (*n* = 60 in total). The eggs were incubated in an incubator (Janeschitz, Hammelburg, Germany) at a constant temperature of 38 °C and constant humidity conditions. After 72 h of incubation, 8–10 ml egg white was extracted using a sterile disposable syringe and an oval opening was cut into the eggshell. The opening was then sealed with Parafilm® (Cole-Parmer, Wertheim, Germany) to protect the chicken embryo from external hazards. The eggs were then returned to the incubator for further incubation.

On the 10th day of their embryological development day, the membranes were placed on the CAM using sterile tweezers. The membranes were positioned near blood vessels but embryo distant. First, native Mucoderm®, native A-PRF and Mucoderm® biofunctionalized with native A-PRF were compared. Second, biofunctionalized Bio-Gide® using i-PRF or hyaluronic acid, biofunctionalized Smartbrane membrane using hyaluronic acid and native Bio-Gide® were applied. The opening in the eggshell was then sealed with Parafilm, and the eggs were placed back in the incubator.

Photodocumentation was performed using a digital microscope (KEYENCE, Neu-Isenburg, Germany). In the Mucoderm® experiments, images were taken at 0 h and 24, 48, 72, 96 and 120 h consequently. A uniform grid of squares with a side length of 500 μm was overlaid on the imaging screen. The membrane under investigation was centered in the grid. Once both focus and brightness were correctly adjusted, images were captured at 30×, 50×, and 100× magnification.

### Extraction of the membranes and histological preparation

The membranes were extracted along with the CAM using sterile scissors, placed on weighing paper (Carl Roth GmbH + Co. KG, Karlsruhe, Germany), and embedded in a cassette (Kabe Labortechnik, Nümbrecht, Germany). Immediately following extraction, the largest blood vessels near the embryo were severed. The embedding cassettes were fixed in Roti-Histofix 4.5% for at least 24 h. The fixed tissue was then trimmed, positioned in embedding capsules, and processed in the embedding machine (Tissue-Tek VIP 5Jr, Sakura Finetek Europe). Subsequently, the specimens were embedded into blocks and sectioned into 5 μm thick slices, which were then mounted on slides.

### Staining

For Hematoxylin-Eosin (HE) staining, the samples were first deparaffinized. They were placed at 1:10 diluted hematoxylin and then rinsed under running water. This was followed by an immersion in eosin, a brief rinse in distilled water, and then sequential treatment in 70%, 96%, and 100% alcohol. The slides were then treated in xylene and mounted using Eukitt mouting medium.

For alpha-smooth muscle actin (SMA) staining, deparaffinization was performed. The samples were then placed in sodium citrate buffer at pH 6 in a steam cooker for 30 min for antigen retrieval. This was followed by a treatment with Peroxidase Block (Dako, Jena, Germany) and DAKO Protein Block (Dako, Jena, Germany). After one hour, HRP anti-mouse polymer (Dako, Jena, Germany) was applied for 30 min. The samples were then treated with DAB (Dako, Jena, Germany), followed by a hematoxylin treatment, rehydration, and mounting using Eukitt.

For CD105 staining, the slides were deparaffinized and treated with 0.1% Triton-X-100 (Sigma-Aldrich, St. Louis, MO, USA) for permeabilization. They were blocked using 5% PBS/BSA (Sigma-Aldrich, St. Louis, MO, USA) and PBS/Goat NS (Dako, Jena, Germany). The slides were then incubated with CD105 anti-chicken antibody (1:750; Biorbyt, Cambridge, England) for 1 h, followed by incubation with alpha rabbit 488 antibody (1:100; Invitrogen, Carlsbad, CA, USA) for 60 min. Finally, the samples were treated with DAPI (1:1000; ThermoFisher, Waltham, MA, USA) and mounted using Fluorescence Mount Medium (Dako S3023; Dako, Jena, Germany).

### Immunohistochemical/ IKOSA® analysis

**Fig. 2:**
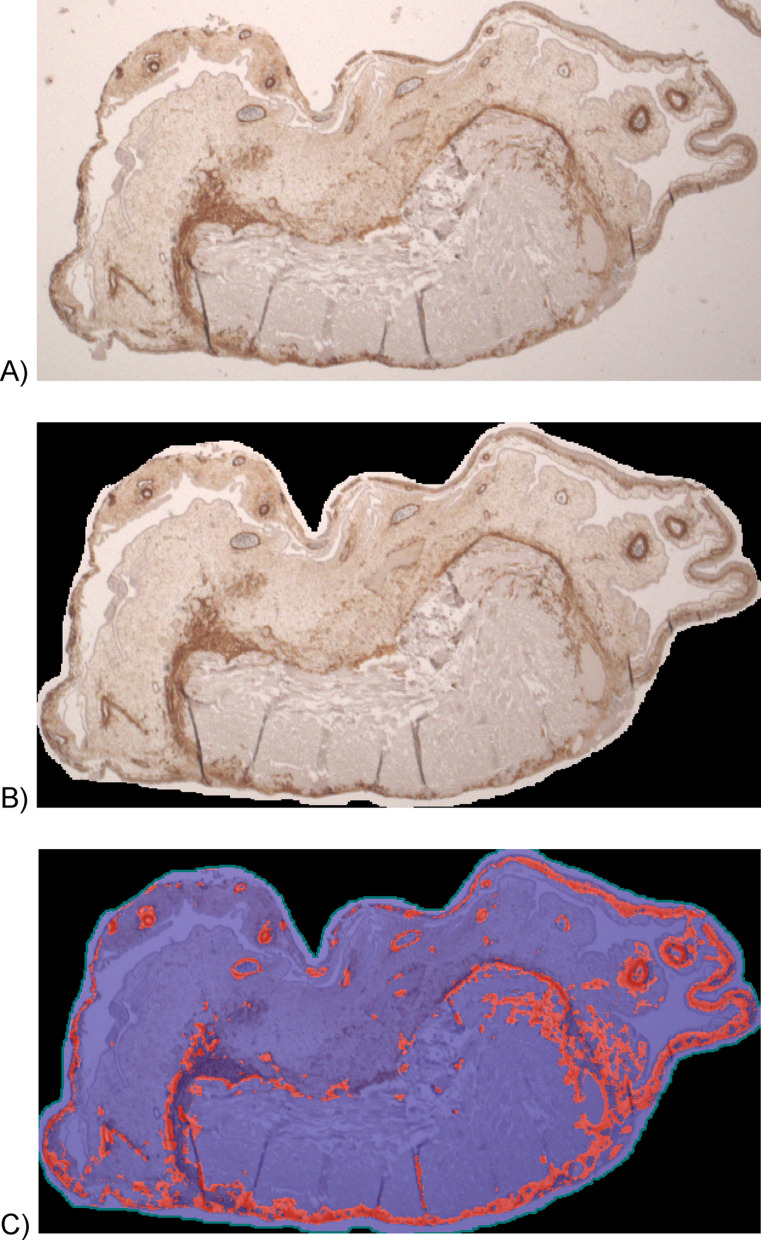
Exemplary analysis of an α-SMA section, (**A**) photographed, (**B**) cropped, (**C**) analysis using BZII-analyzer

The histological sections stained with α-SMA and CD105 were analyzed using the Keyence Biorevo BZ-9000 microscope (Keyence, Neu-Isenburg, Germany) and the accompanying BZII-Viewer and BZII-Analyzer software (Brightfield HF and Phako, microscope position 2 Plan Apo Na.10; Keyence, Neu-Isenburg, Germany). The functions “Hybrid Cell Count” and “Brightfield & Single Extraction” were used to quantify the percentage of stained tissue as an area ratio. Additionally, a manual count of the vessels was performed in the HE-stained sections (Fig. 2).

**Fig. 3:**
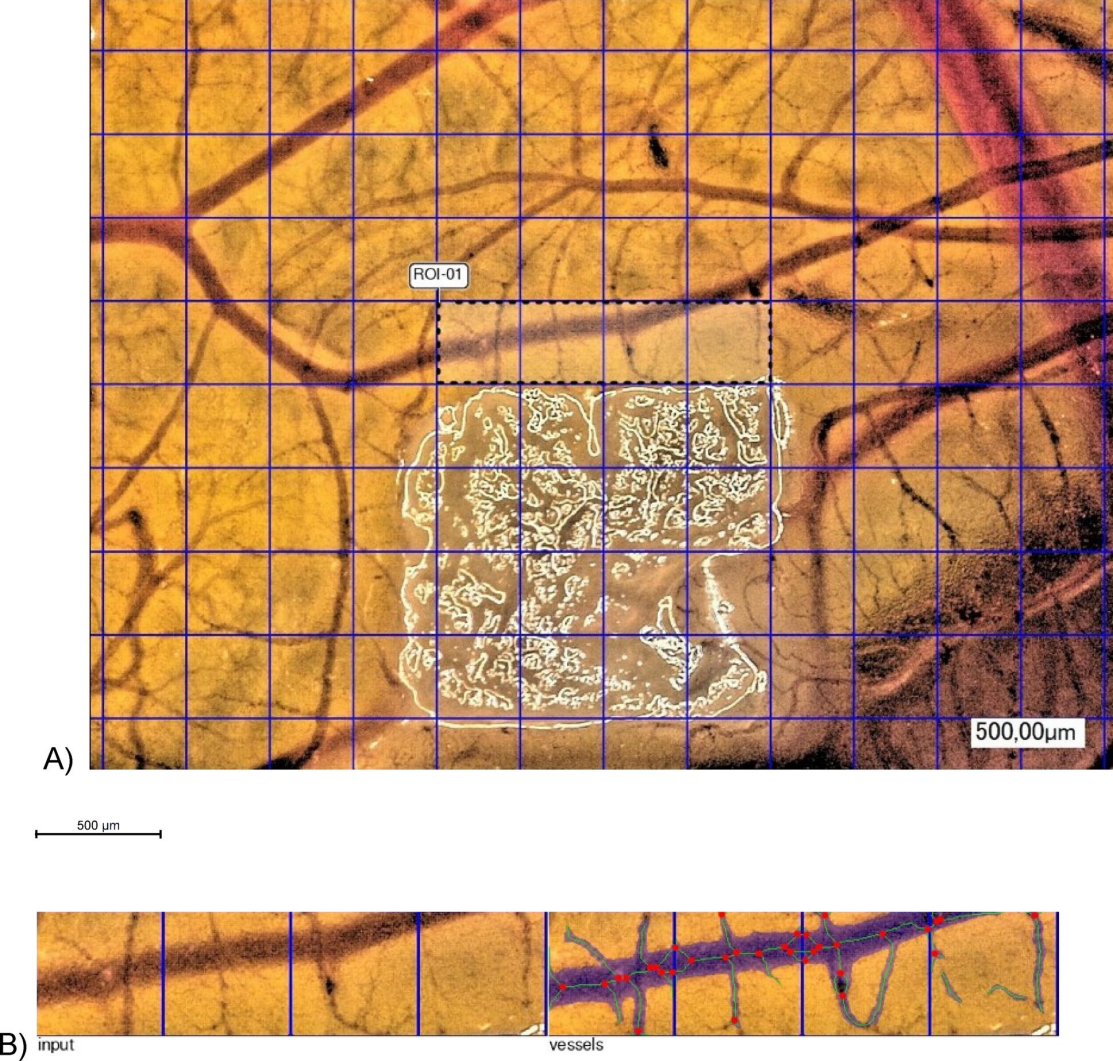
Example analysis using IKOSA® CAM Assay Software; (**A**) selected ROI (Region of Interest), (**B**) left: input image; right: output image; red dots: branching points; blue shading: detected vessels; green lines: vessel paths

The images captured by the digital microscope were analyzed using the IKOSA® CAM Assay software (©KML Vision GmbH, version 3.2). The images were uploaded to the respective IKOSA® database and a standard region of interest (ROI) measuring 4 × (500 × 500) µm was selected. The software then analyzed the total area, length and thickness of the identified vessels, as well as the number of branching points (Fig. 3).

### Statistical analysis

The statistical analysis was performed using IBM SPSS Statistics (Version 27; IBM, Ehningen, Germany). Data are reported as mean ± SD. Given the exploratory character of this study and the limited sample size, no correction for multiple comparisons was applied. *P*-values should therefore be interpreted descriptively. First, normality of the data was tested using the Shapiro-Wilk test and the Kolmogorov-Smirnov test. *P*-values > 0.05 were normally distributed. Normally distributed data sets were then analyzed using the t-test. For non-normally distributed data, the Mann-Whitney U test was applied. Results were visualized as boxplots.

## Results

### A-PRF significantly promotes angiogenesis in ovo for up to 96 h

Biofunctionalization of MM with A-PRF significantly enhanced angiogenic potential compared to the native MM, as shown by Hematoxylin-Eosin (HE) staining after 48 h (*p* = 0.045), 72 h (*p* < 0.001) and 96 h (*p* = 0.002). At 24 and 120 h vessel counts for biofunctionalized MM were descriptively higher than native MM, but these differences were not significant. The IKOSA® analysis confirmed these findings, showing a descriptive increase in total vessel area, length, thickness, and number of branching points after 48 and 72 h for biofunctionalized MM compared to native MM. After 72 h, in particular, the total vessel area was significantly larger in the biofunctionalized MM (*p* = 0.0497). At 96 h, descriptive differences favoring the biofunctionalized MM persisted for vessel area and thickness. However, after 120 h, the IKOSA® analysis descriptively showed consistently better values for the native MM.

Biofunctionalization was also performed with i-PRF. Hematoxylin-Eosin (HE) staining revealed significantly larger vessel growth for BM with i-PRF after 24 h compared to BM with hyaluronic acid (HA) (*p* = 0.018), native Mucoderm® membrane (MM) (*p* = 0.036) and native A-PRF (*p* = 0.031). After 48 h, BM with i-PRF continued to show greater angiogenic potential compared to native MM (*p* = 0.040). These findings were confirmed by the IKOSA® analysis, which indicated increased vessel length for BM with i-PRF after 48 h relative to native A-PRF (*p* = 0.047). Compared to native MM, BM with i-PRF also showed more branching points after 48 h (*p* = 0.049) and after 72 h, demonstrated significantly better values for parameters total area (*p* = 0.007), length (*p* = 0.018), and thickness (*p* = 0.008). Notably, no significant differences were observed when comparing biofunctionalized BM with i-PRF to native BM in α-SMA, CD105 and HE staining after 48 and 72 h, though descriptive differences were identified. The IKOSA® analysis also showed descriptively higher values across nearly all parameters and time points for BM with i-PRF compared to the native variant. In a direct comparison of native membranes, HE staining showed a significant difference between native BM and native MM. Native BM exhibited a significantly higher pro-angiogenic potential at 24 h (*p* = 0.012). In both the IKOSA® analysis, and α-SMA and CD105 staining, native BM was generally superior to native MM, although no significant differences were evident. After 24 h, native BM also demonstrated significantly greater angiogenic potential compared to SM + HA (*p* = 0.034), BM + HA (*p* < 0.001), and native A-PRF (*p* = 0.002), while native MM showed a significantly greater pro-angiogenic effect than SM + HA (*p* = 0.038).

Native A-PRF also proved beneficial. After 24 h, it significantly outperformed SM + HA (*p* < 0.001) in terms of angiogenic effect, and after 48 h, was also significantly superior to native MM (*p* = 0.030). At 72 h, native A-PRF showed a significantly higher vessel count compared to native MM (*p* = 0.005) and native BM (*p* = 0.045).

### Hyaluronic acid significantly promotes angiogenesis in ovo after 48 h and 72 h

At 24 h, membranes combined with hyaluronic acid (SM + HA and BM + HA) showed significantly reduced vessel formation compared to native membranes and those biofunctionalized with PRF. Specifically, the native Bio-Gide® membrane (*p* = 0.034), native A-PRF (*p* < 0.001), native Mucoderm® membrane (*p* = 0.038), and MM biofunctionalized with A-PRF (*p* = 0.012) all exhibited significantly higher vessel counts than the Smartbrane membrane biofunctionalized with hyaluronic acid. Furthermore, native BM (*p* < 0.001), BM biofunctionalized with i-PRF (*p* = 0.018), and MM + A-PRF (*p* = 0.024) demonstrated a greater pro-angiogenic effect than BM biofunctionalized with HA (BM + HA).

**Fig. 4:**
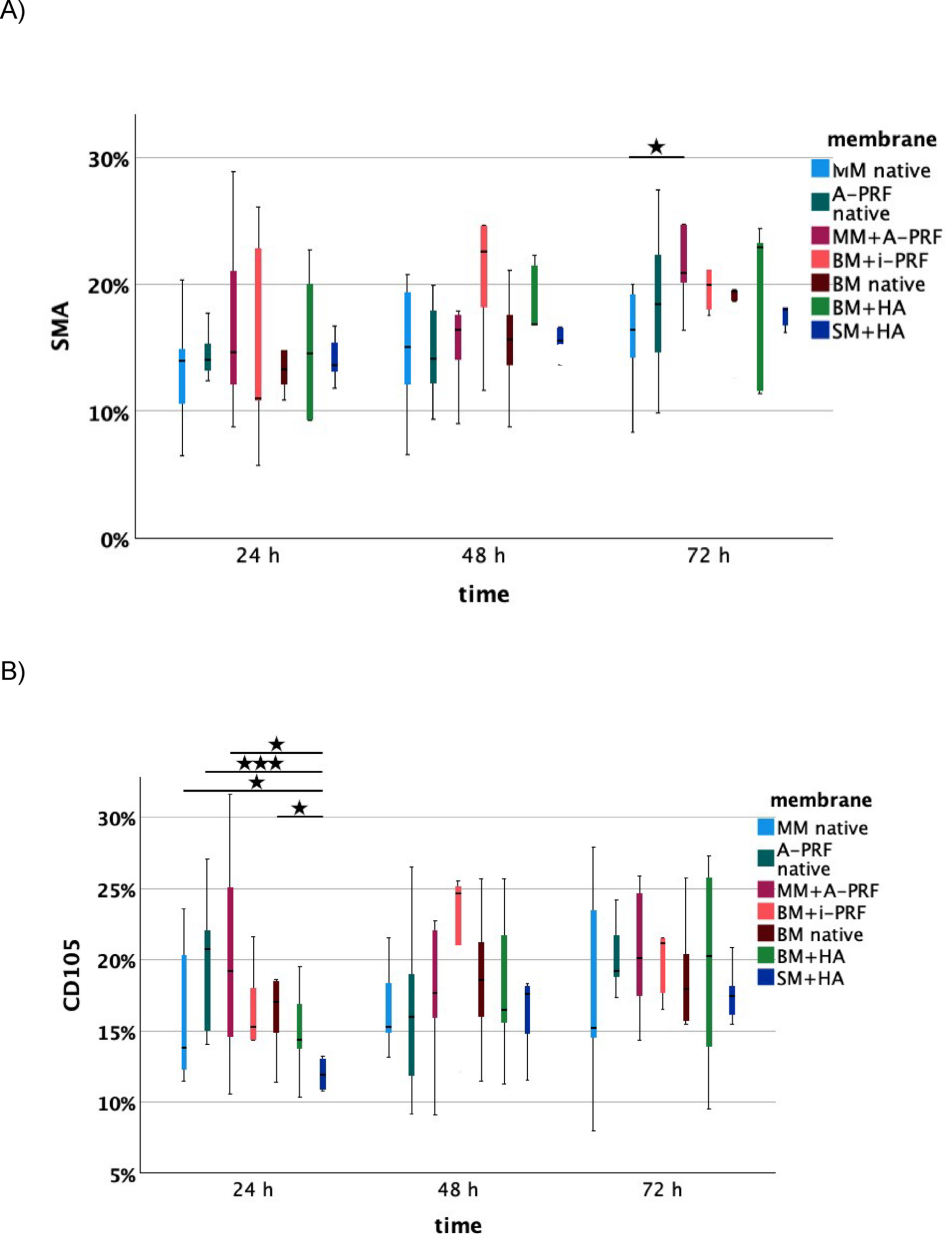

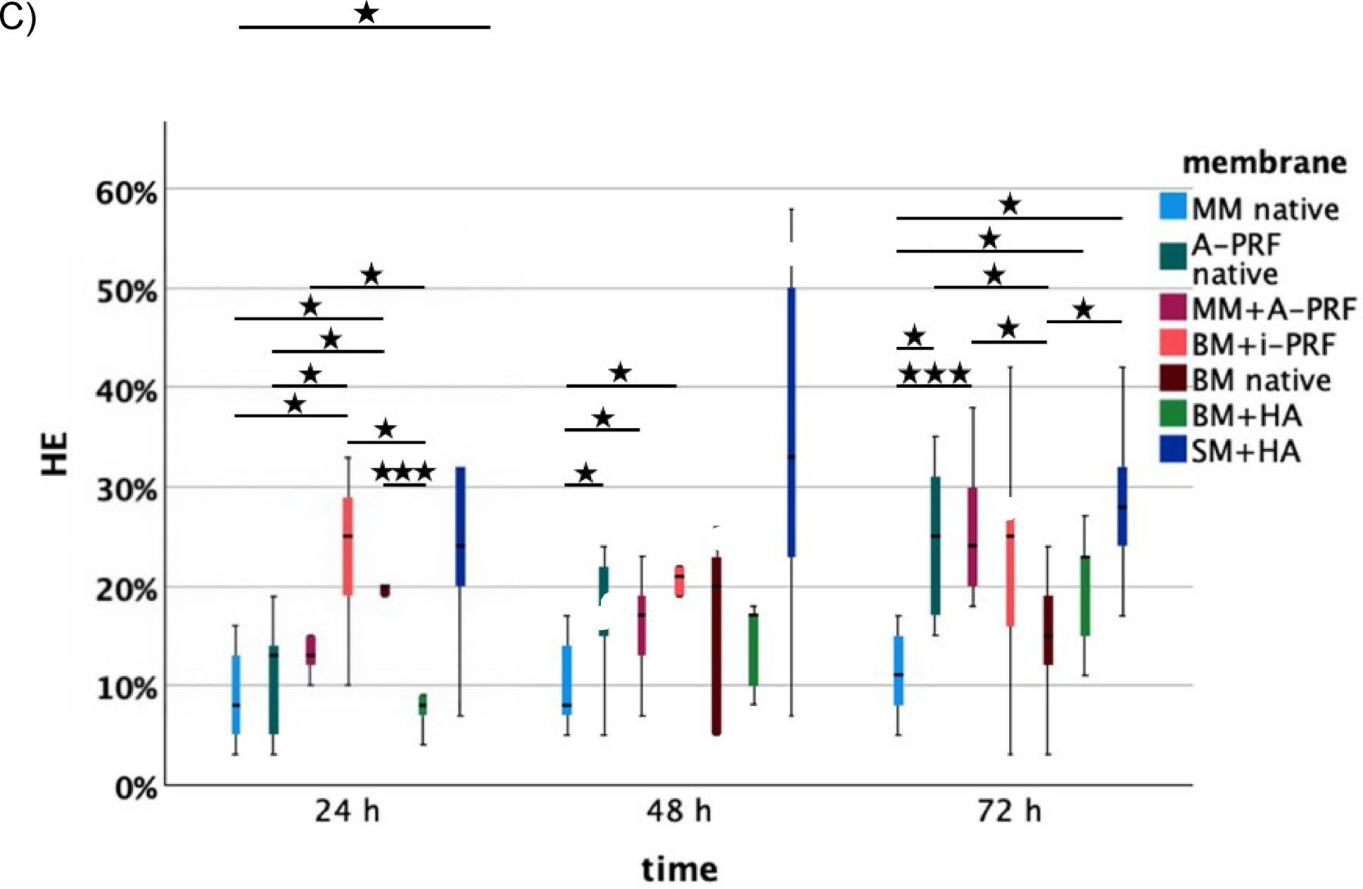
Analysis of (**A**) α-SMA staining, (**B**) CD105 staining, and (**C**) HE staining for each membrane (MM native: native Mucoderm®, A-PRF native, MM + A-PRF: Mucoderm® with A-PRF, BM + i-PRF: Bio-Gide® with i-PRF, Bio-Gide® native, BM + HA: Bio-Gide® with HA, SM + HA: Smartbrane membrane with HA) after 24 h, 48 h, and 72 h. Lines indicate significant differences (based on t-tests), with significance levels denoted as follows: *p < 0.05, **p < 0.01, ***p < 0.001

**Fig. 5:**
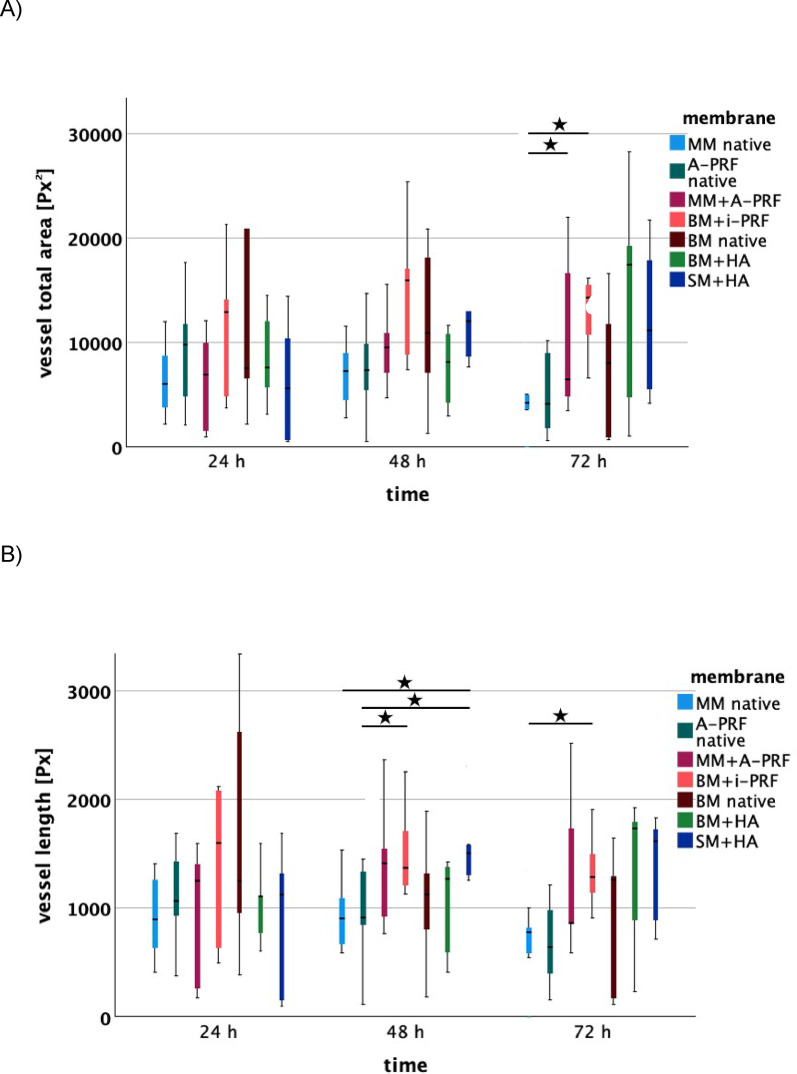

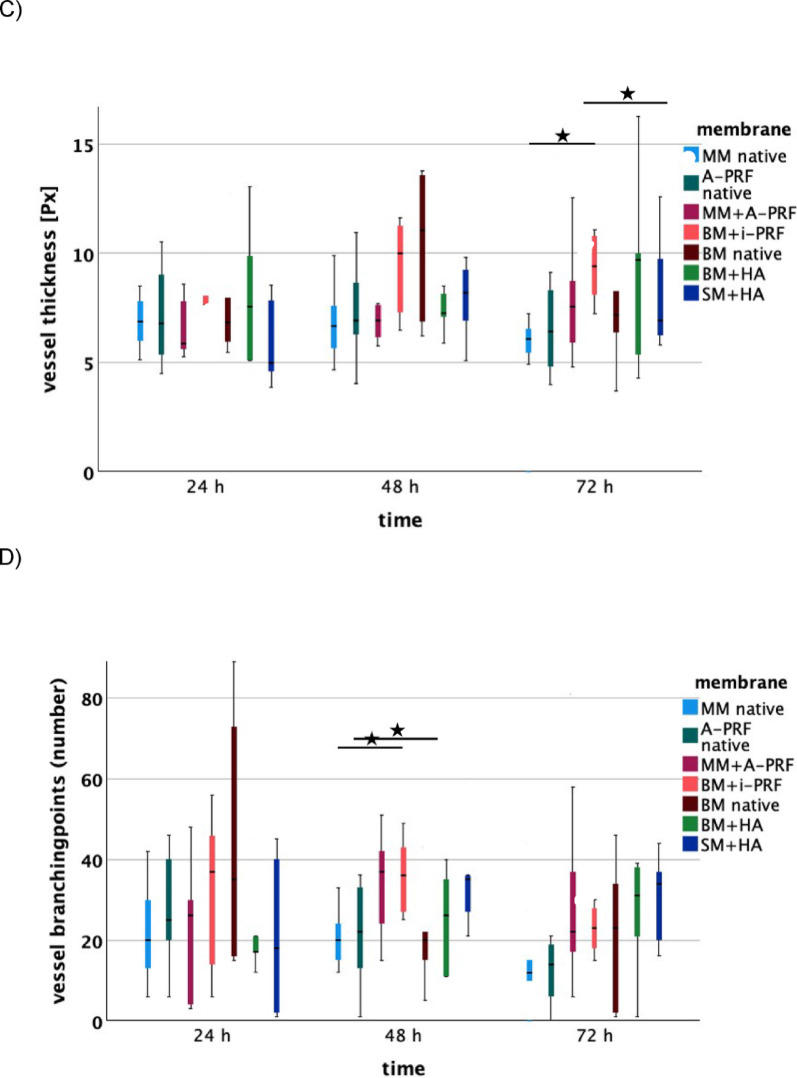
IKOSA® analysis of parameters (**A**) total area in pixel^2^ [Px^2^], (**B**) length in pixel [Px], (**C**) thickness in pixel [Px], and (**D**) number of branching points for the respective membranes (MM native: native Mucoderm®, A-PRF native, MM + A-PRF: Mucoderm® with A-PRF, BM + i-PRF: Bio-Gide® with i-PRF, Bio-Gide® native, BM + HA: Bio-Gide® with HA, SM + HA: Smartbrane membrane with HA) after 24 h, 48 h, and 72 h. Lines indicate significant differences (based on t-tests), with significance levels denoted as follows: *p < 0.05, **p < 0.01, ***p < 0.001

However, after 48 h, the angiogenic potential of hyaluronic acid became more apparent. SM + HA showed a significantly greater vessel length in the IKOSA® analysis compared to native MM and native A-PRF. At 72 h, SM + HA exhibited significantly greater vessel growth in immunohistochemical analysis compared to native MM (*p* = 0.010) and native BM (*p* = 0.034). Additionally, BM + HA showed significantly more vessels than native MM (*p* = 0.036) (Figs. 4, 5).

Overall, the CAM assay demonstrated that PRF-based biofunctionalization markedly enhances the angiogenic potential of collagen membranes in ovo, with clear time-dependent effects. A-PRF significantly increased vascularization of Mucoderm® membranes particularly between 48 and 96 h, while i-PRF showed an early and robust pro-angiogenic effect, especially on Bio-Gide® membranes, outperforming native membranes across multiple quantitative vascular parameters. Native Bio-Gide® membranes generally exhibited higher intrinsic angiogenic activity than native Mucoderm®, and native A-PRF alone also showed a strong pro-angiogenic effect at early and intermediate time points. In contrast, hyaluronic acid initially reduced angiogenesis at 24 h but demonstrated a delayed stimulatory effect at 48 and 72 h. Collectively, these findings highlight PRF—especially i-PRF—as a potent and early enhancer of angiogenesis, whereas hyaluronic acid exerts a later, time-dependent pro-angiogenic influence.

## Discussion

This experimental study demonstrates the pro-angiogenic potential of biofunctionalizing various collagen matrices with A-PRF, i-PRF, and HA by using the CAM assay to assess vascularization and angiogenesis in ovo. The core finding is that combining collagen matrices (CM) with A-PRF significantly enhanced angiogenesis in ovo, with effects sustained up to 96 h. However, their pro-angiogenic potential and clinical relevance remain exploratory at this stage. The findings support biofunctionalization with A-PRF as an approach to improve techniques in guided bone regeneration (GBR) and guided tissue regeneration (GTR). While biofunctionalization with i-PRF also demonstrated beneficial pro-angiogenic effects within the 24–72 h timeframe, no significant difference in efficacy between A-PRF und i-PRF in promoting angiogenesis in ovo was evident. Clinically, both A-PRF and i-PRF could be considered effective options for prevascularizing collagen membranes. In summary, it must be emphasized that the present study is a purely in ovo investigation without any in vivo or clinical correlation. The study has several limitations, for example related to the constraints of the model itself, the short observation period, and the relatively small sample size.

Biofunctionalization with HA yielded further insights. A significant increase in angiogenesis was observed at 48- and 72-h following HA treatment. Previous studies by our research group have shown that PRF-based biofunctionalization of collagen membranes can enhance angiogenesis. For example, Blatt et al. (2020) demonstrated increased vessel number per mm^2^, vascular density, and branching points in biofunctionalized membranes (Mucoderm®, Jason®, Collprotect®) after 24 h, both in vivo and in vitro. Immunohistochemical staining for α-smooth muscle actin (α-SMA) and CD105 confirmed significantly higher pro-angiogenic activity compared to native controls [13]. Similarly, a study using PRF on Symbios® membranes reported increased vessel formation and branching points after 72 h compared to native YSM membranes, although differences between PRF-treated and native membranes were not always statistically significant [14]. The results of this provide additional evidence that the biofunctionalization of CM with A-PRF can increase vascularization and angiogenesis in ovo, extending prior findings by demonstrating increased angiogenic activity at 48 and 96 h with A-PRF, suggesting a sustained effect. However, no definitive conclusions can be drawn for the 120-h time point, as discrepancies were noted between immunohistochemical and IKOSA® software analyses. While both methods are established tools for assessing angiogenesis, each has limitations [13, 14, 38, 39]. Image quality (e.g., glare, reflections), staining inconsistencies and manual histological evaluation may have affected the reliability and comparability of results.

Biofunctionalization of BM with i-PRF also demonstrated early angiogenic potential (24–72 h). The observed differences in performance between i-PRF and A-PRF may be related to their distinct release kinetics and growth factor profiles, as reported in previous studies. Choukroun et al. (2017) and Miron et al. (2019) reported, that i-PRF contains higher platelet and leukocyte concentrations than A-PRF in the early phase and is associated with a more rapid and pronounced early release of angiogenesis-related growth factors, including VEGF and TGF-β1, compared with A-PRF [40, 41]. Conversely, cumulative long-term growth factor release has been reported to be lower for i-PRF than for A-PRF, with the exception of IGF-1 [42]. In contrast, A-PRF exhibits a more sustained release profile, with VEGF release surpassing that of i-PRF after one day and TGF-β release after approximately three days, and significantly higher cumulative levels of VEGF, TGF-β, PDGF-BB, and PDGF-AA over seven days [42, 43]. These literature-based findings are consistent with the temporal angiogenic patterns observed in the present CAM assay and suggest that i-PRF may be particularly suited for short-term angiogenesis-oriented applications, whereas A-PRF may offer advantages for longer-term regenerative processes. However, as growth factor concentrations were not directly quantified in this study, these mechanistic explanations remain inferential. Future studies incorporating direct growth factor measurements and extended observation periods beyond 96–120 h are warranted to more robustly elucidate long-term angiogenic and regenerative efficacy.

In the present study, i-PRF biofunctionalization of BM promoted angiogenesis between 24 and 72 h; however, similar to A-PRF, no clear superiority was observed within this timeframe. These findings are consistent with those of Schröger et al. (2024), who likewise reported no significant differences between various PRF modifications for prevascularizing collagen membranes [44]. A previous investigation by our group further demonstrated that i-PRF failed to enhance angiogenesis when used to biofunctionalize acellular porcine collagen membranes, whereas native membranes exhibited stronger proangiogenic effects for up to 72 h. These differences were likely attributable to variations in membrane composition (e.g., NovoMatrix™: Collagen I/VI; Bio-Gide®: Collagen I/III) [31].

Material properties strongly influence PRF integration [36, 45], as the penetration of i-PRF and its interaction with the membrane depend largely on the material’s characteristics. Al-Maawi et al. (2019) demonstrated that i-PRF enhances the bioactivity of collagen materials, but penetration varies by membrane type, with full infiltration into Mucograft®, partial penetration in Bio-Gide® and Mucoderm®, superficial penetration in Collprotect® and none in BEGO® membranes. Structural properties, such as dense, tightly woven fibers (e.g., in Mucoderm®), likely limit PRF uptake and vascular infiltration, potentially explaining reduced angiogenic effects observed at early time points. There were also differences in the i-PRF centrifugation protocol (600 rpm, 8 min) (Duo Centrifuge, Process for PRF, Nice, France) [46]. The compact structure and larger, tightly interwoven fibers of MM, as evidenced by scanning electron microscopy, may explain this phenomenon [46]. This structural difference could also affect the infiltration of blood vessels in the chorioallantoic membrane (CAM) assay, potentially explaining the lower angiogenic potential observed at 24 h.

The native variants performed well in this study, including native A-PRF, consistent with our previous findings [13, 14], however they are not directly comparable to biologized variants in terms of sustained effects due to structure, thickness, length and crosslinking.

While the angiogenic effect of HA proved to be less pronounced than that of A-PRF or i-PRF a significant influence was evident at 48 and 72 h. These finding complements those of Kyyak et al. (2022), who reported increased angiogenesis when bovine bone substitute materials were biofunctionalized with HA, with improvements in vessel number, length, area and branching after 144 h (day 13 of chick embryo development) [26]. Despite its apparently more limited effect, HA offers several clinical advantages. It eliminates the need for blood collection, centrifugation, and PRF processing, thereby simplifying application, reducing cost and personnel requirements, and minimizing patient burden — which is especially beneficial for anxious patients. Longer observation periods (up to 120 h) will be needed to fully evaluate HA’s angiogenic potential and clinical utility.

## Conclusion

This study evaluated the angiogenic effects of collagen membranes biofunctionalized with A-PRF, i-PRF, and HA, using the CAM assay in ovo supported by both immunohistochemical and AI-assisted (IKOSA®) analyses. To the best of our knowledge, for the first time an extended observation period (up to 120 h) in ovo and a direct comparison of hyaluronic acid and platelet-rich fibrin for membrane biofunctionalization was performed. A-PRF showed sustained angiogenic activity up to 96 h in the CAM model, while i-PRF produced similar effects between 24 and 72 h, without clear superiority. HA also induced angiogenic responses, particularly at 48 and 72 h. These findings suggest differing early vascular effects among biofunctionalization approaches; however, their translational relevance is limited by the CAM model’s lack of a mineralized bone environment, mature immune system, and mechanical loading. Thus, the results should be interpreted as preliminary and restricted to early angiogenesis. HA may represent a cost-efficient alternative worth further investigation, but any potential advantages require confirmation in appropriate in vivo models. Longer observation periods and subsequent mammalian studies are needed to clarify the long-term angiogenic profiles and comparative effectiveness of A-PRF, i-PRF, and HA.

## Data Availability

No datasets were generated or analysed during the current study.

## Abbreviations

°C: Degree celsius
%: Percent
3D: Three-dimensional
A-PRF: Advanced PRF
α-SMA: Anti-alpha smooth muscle actin antibody (1A4)
µm: Micrometer
BM: Bio-Gide®
BM + HA: Bio-Gide® biofunctionalized with hyaluronic acid
BM + i-PRF: Bio-Gide® biofunctionalized with i-PRF
CD105: Immunofluorescence anti-chicken antibody
CM: Collagen membranes
cm: Centimeter
cm^2^: Square centimeters
Dako: Marked polymer-HRP anti mouse
e.g.: Exempli gratia
G: Gauge
GBR/GTR: Guided bone- and tissue regeneration
h: Hours
HA: Hyaluronic acid
HE: Hematoxylin-eosin
i-PRF: Injectable PRF
min: Minute
ml: Milliliter
mm: Millimeter
mm^2^: Square millimeter
MM: Mucoderm® membrane
MM + A-PRF: Mucoderm® biofunctionalized with A-PRF
n: Sample number
p: Likelihood
PBS: Phosphate buffered salt solution
ph: pH value
PRF: Platelet-rich-fibrin
ROI: Region of Interest
rpm: Revolutions per minute
s: Second
SM + HA: Smartbrane membrane biofunctionalized with hyaluronic acid
TGF-β: Transforming growth factor-beta
